# Intramuscular oxytocin versus oxytocin/ergometrine versus carbetocin for prevention of primary postpartum haemorrhage after vaginal birth: study protocol for a randomised controlled trial (the IMox study)

**DOI:** 10.1186/s13063-018-3109-2

**Published:** 2019-01-03

**Authors:** Helen van der Nelson, Stephen O’Brien, Erik Lenguerrand, Elsa Marques, Mary Alvarez, Michelle Mayer, Sara Burnard, Dimitrios Siassakos, Timothy Draycott

**Affiliations:** 1 Translational Health Sciences, Bristol Medical School, University of Bristol, Bristol, UK; 2 Clinical Research Fellow in Obstetrics & Gynaecology, Translational Health Sciences, Bristol Medical School, University of Bristol, Canynge Hall, 39 Whatley Road, Bristol, BS8 2PS UK; 3 Women & Children’s Directorate, North Bristol NHS Trust, Bristol, UK; 4 NIHR Clinical Research Network; West of England, Bath, UK

**Keywords:** Postpartum haemorrhage, Prevention, Primary, Oxytocin, Oxytocin/ergometrine, Carbetocin

## Abstract

**Background:**

Postpartum haemorrhage remains a major cause of maternal mortality and morbidity worldwide. Active management of the third stage of labour reduces the risk of postpartum haemorrhage. Oxytocin and oxytocin/ergometrine are commonly used in the UK, with oxytocin/ergometrine being more effective at preventing moderate, but not severe, blood loss. Many guidelines specifically recommend using oxytocin for all vaginal births, as it is associated with fewer adverse events. However, a survey conducted by the Southmead Hospital Maternity Research Team revealed that 71.4% of UK obstetric units still routinely use oxytocin/ergometrine. Carbetocin is a newer medication that may be as effective but has fewer side effects. No studies have directly compared all three medications.

**Methods:**

The IMox study aims to determine the most effective, acceptable and cost-effective drug for primary prevention of postpartum haemorrhage following vaginal birth. The IMox study is a prospective, multi-centre, double-blind, randomised trial directly comparing oxytocin, oxytocin/ergometrine and carbetocin given intramuscularly for the prevention of postpartum haemorrhage in the third stage of labour. The primary effectiveness outcome is the use of an additional uterotonic drug. Secondary effectiveness outcomes reflect maternal morbidity and mortality within the immediate postpartum period. Participant questionnaires and subjective reporting of side effects will be used to evaluate maternal acceptability. Maternal quality of life utilities will be collected antenatally, and on days 1 and 14 after birth to enable a cost-effectiveness assessment of each studied drug.

Participants will be pregnant women planning a vaginal birth in six hospitals in England. Participants will be approached and invited to provide consent to participate from 20 weeks gestation until in established labour. A complete sample of 5712 participants (1904 per arm) providing data for the primary outcome will allow for a robust determination of efficacy between all three study drugs. Data will be collected until participants are discharged from the hospital and on postnatal days 1 and 14 regardless of location. All analyses will be on a modified intention-to-treat basis, and additionally repeated on a per protocol basis. Data collection commenced in Feburary 2015 and was completed in August 2018.

**Discussion:**

This study is the first to directly compare oxytocin, oxytocin/ergometrine and carbetocin in the same population for the prevention of postpartum haemorrhage following vaginal birth. Furthermore, this study will be the first to directly compute health economic outcomes from such a three-way comparison. This study is limited to using short-term outcomes, and so will not provide evidence for important outcomes such as long-term maternal psychological well-being and time to next conception.

**Trial registration:**

ClinicalTrials.gov, NCT02216383. Registered on 18 August 2014. EudraCT, 2014-001948-37. Registered on 23 September 2014. ISRCTN, ISRCTN10232550. Retrospectively registered on 6 March 2018).

**Electronic supplementary material:**

The online version of this article (10.1186/s13063-018-3109-2) contains supplementary material, which is available to authorized users.

## Background

Primary postpartum haemorrhage (PPH), defined as a loss of ≥500 ml of blood from the genital tract within 24 h of delivery, remains a major cause of maternal morbidity and mortality worldwide, accounting for more than a quarter of all global maternal deaths [1]. In addition to the significant risk of immediate adverse events, PPH is significantly associated with long-term physical and psychological morbidity [2]. The need for treatments such as additional uterotonic drugs, blood transfusion, operative procedures including hysterectomy and prolonged hospital stay affect not just the woman experiencing the haemorrhage, but also her family and the health service, which incurs additional costs. Approximately 70% of PPHs are caused by inadequate uterine contraction after delivery of the placenta (uterine atony) [3]. Prophylactic administration of a uterotonic drug in the third stage of labour, together with early cord clamping and controlled cord traction, reduces the incidence of primary PPH by 66%, when compared with physiological management [4]. The majority of women who experience PPH have no risk factors [5], and it is therefore recommended that this active management of the third stage of labour (AMTSL) is routinely offered to all labouring women [5–7].

There are several prophylactic uterotonic agents available for use, including oxytocin, oxytocin/ergometrine and carbetocin. Oxytocin and oxytocin/ergometrine are commonly used for vaginal deliveries, while carbetocin is presently only licensed for use at caesarean section. Carbetocin is a synthetic analogue of oxytocin which provides a longer duration of action than oxytocin (half-life 85–100 min versus 3–4 min) [8].

Although there are no trials directly comparing all three of oxytocin, oxytocin/ergometrine and carbetocin for prophylaxis of PPH after vaginal birth, a Cochrane network meta-analysis (NMA) on this subject was published in 2018 [9]. This NMA demonstrated rates for PPH ≥ 500 ml of 10.5%, 7.2% and 7.6% for oxytocin, oxytocin/ergometrine and carbetocin respectively, and rates of PPH ≥ 1000 ml of 3.6%, 2.8% and 2.5% respectively. Additionally, the NMA demonstrated higher rates of adverse outcomes following the use of oxytocin/ergometrine than both oxytocin and carbetocin (hypertension 1.2%, 0.7% and 0.6% respectively and vomiting 1.9%, 0.5% and 0.6% respectively).

Since publication of this NMA, the results of a large multi-national non-inferiority clinical trial in 30,000 women of oxytocin versus carbetocin for prophylaxis of PPH have been published by Widmer et al. [10]. This trial demonstrated non-inferiority of carbetocin relative to oxytocin for the prevention of PPH ≥ 500 ml (relative risk (RR) 1.01; 95% confidence interval (CI), 0.95 to 1.06). The use of additional uterotonic agents and interventions to stop bleeding and the occurrence of adverse effects were not shown to differ significantly between the two groups.

The trial was not sufficiently powered to demonstrate non-inferiority for PPH ≥ 1000 ml.

Taken together, these recent results suggest that carbetocin may be as effective as (or potentially more effective than) oxytocin, and associated with fewer adverse events than oxytocin/ergometrine.

Current national and international guidelines advocate the use of intramuscular oxytocin as the prophylactic uterotonic agent of choice in the third stage of labour for low-risk women delivering vaginally [5–7, 11]. However, an unpublished telephone survey of all obstetric units in the UK in October 2013 conducted by our team found that 71% of units still use oxytocin/ergometrine for normotensive women having a vaginal birth despite these recommendations. Many units volunteered that their local departmental audits had shown increases in their PPH rates following a switch to oxytocin in line with national guidance, and that they therefore reverted to oxytocin/ergometrine despite its known side effects. The care being provided to women on a national scale is clearly suboptimal in this respect, which suggests that an alternative prophylactic uterotonic agent may be needed.

Very few studies have investigated the cost-effectiveness of carbetocin. The need for new studies investigating the cost and cost consequences of carbetocin was highlighted in a Cochrane review [12]. This additional information is crucial, particularly as carbetocin is more expensive at UK list price (£17.64 versus £0.91 for oxytocin and £1.38 oxytocin/ergometrine), and any additional benefits or resource use would need to be weighed against the higher acquisition price.

While the recent Cochrane NMA and the study by Widmer et al. add substantial weight to the case that carbetocin may be as effective as (and potentially more effective than) oxytocin and associated with fewer adverse events than oxytocin/ergometrine, the resistance to bring practice into line with current guidelines (at least within the UK) demonstrates the need for primary direct trial evidence for the clinical effectiveness, maternal acceptability and cost-effectiveness of these commonly used drugs.

## Methods/design

### Aims

The primary study aims are to determine if:

- Carbetocin is at least as effective as oxytocin/ergometrine
- Carbetocin is more effective than oxytocin
- Oxytocin/ergometrine is more effective than oxytocin

The secondary study aims are to establish whether:

- Carbetocin is associated with fewer adverse events than oxytocin/ergometrine and oxytocin
- Carbetocin is a cost-effective alternative to oxytocin/ergometrine and oxytocin

### Trial design and justification of comparisons

**Fig. 1:**
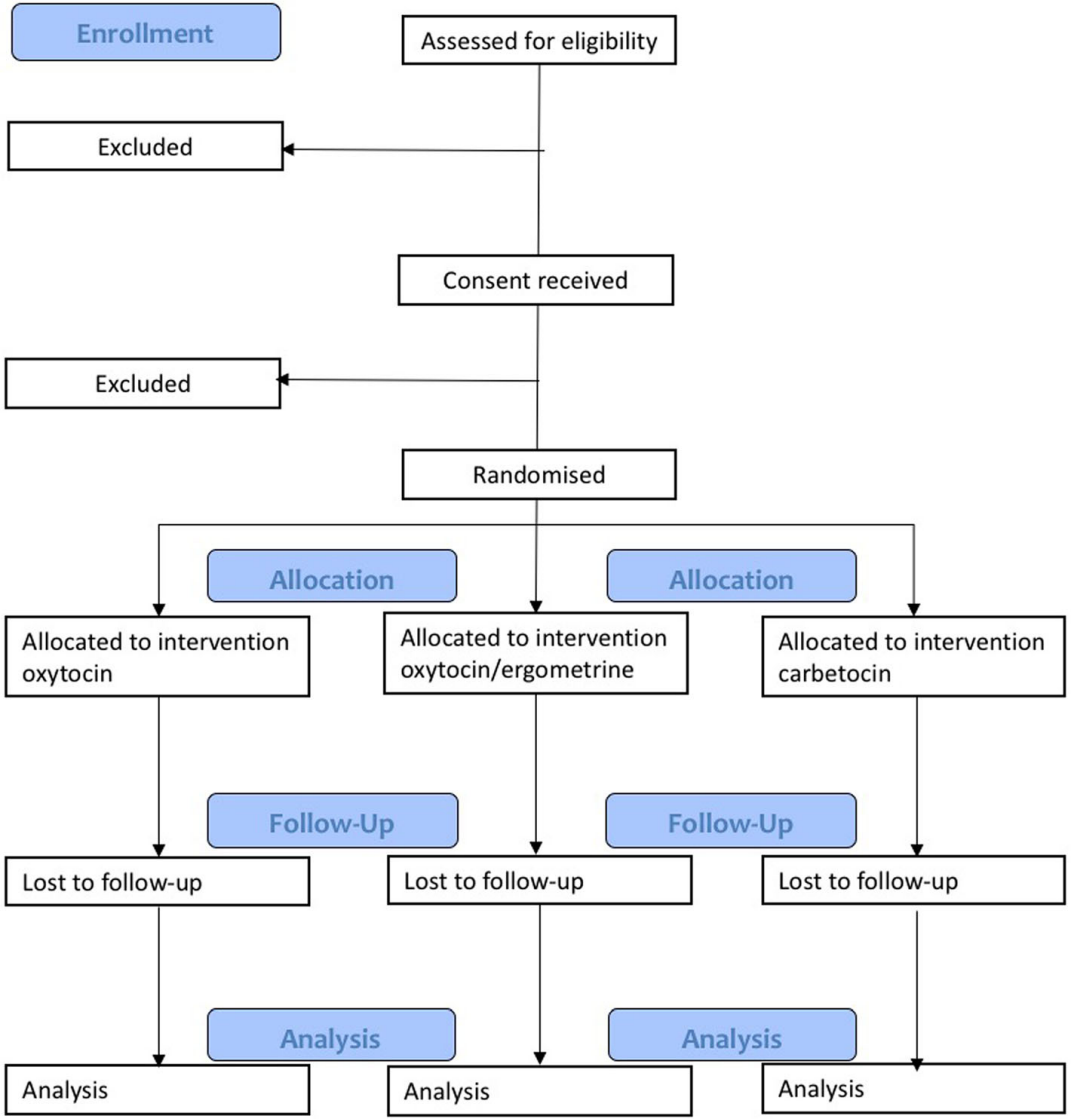
CONSORT diagram of the IMox study

The IMox study is a multi-centre, double-blinded, randomised trial to compare oxytocin, oxytocin/ergometrine and carbetocin given intramuscularly after vaginal birth for prevention of primary PPH. Drugs will be compared in terms of clinical effectiveness, maternal acceptability and cost. Participants will be randomised to parallel arms in a 1:1:1 ratio. A Consolidated Standards of Reporting Trials (CONSORT) diagram of the IMox study is given in Fig. 1.

A non-inferiority comparison between carbetocin and oxytocin/ergometrine has been chosen, as oxytocin/ergometrine is associated with greater maternal side effects. Should carbetocin be non-inferior to oxytocin/ergometrine for the primary outcome and be associated with fewer maternal side effects, it is likely to be superior as a prophylactic uterotonic drug. Superiority comparisons will be drawn between oxytocin/ergometrine and oxytocin as well as carbetocin and oxytocin.

### Participants

Women aged ≥ 18 years with a live singleton pregnancy who have a vaginal birth (spontaneous or instrumentally assisted) at any gestation are eligible to participate. The exclusion criteria are as follows: women with known or suspected antenatal hypertensive disorders, intrapartum hypertension (defined as a single systolic blood pressure ≥ 160 mmHg, or two consecutive blood pressures of ≥ 140 mmHg systolic or ≥ 90 mmHg diastolic taken 30 min apart), antepartum haemorrhage of ≥ 50 ml, confirmed maternal coagulation disorder or intrauterine fetal death in the current pregnancy, women who would decline blood products, women with severe peripheral vascular, cardiac or hepatic disease, women with epilepsy, women with an allergy or sensitivity to any of the ingredients in any of the study drugs, women unable to read and write English sufficiently as to preclude completion of maternal questionnaires and caesarean section.

### Setting

The sites will be six maternity units across the south-west and centre of England. These include teaching hospitals and district general hospitals.

### Intervention

A single dose intramuscular injection of the study drug (10 IU oxytocin or 500 μg/5 IU oxytocin/ergometrine or 100 μg carbetocin) will be given immediately after the vaginal birth of the baby and clamping of the umbilical cord.

### Outcomes

#### Primary clinical outcome

The primary outcome measure is the proportion of women requiring additional uterotonic drugs after administration of the study drug. This primary outcome measure was chosen over severity of PPH for the following reasons: estimation of obstetric blood loss is known to be inaccurate regardless of the mode of estimation [13, 14]; vaginal or cervical tears contribute to the incidence and severity of PPH without any role of uterine atony; lastly the administration of several additional uterotonic drugs may in some cases be used to successfully avoid blood loss of ≥ 500 ml. This would not be captured with a primary outcome of PPH incidence, which would not account for the clinically important (and costly) administration of additional uterotonics.

#### Secondary outcomes

##### Secondary effectiveness outcomes

Secondary effectiveness outcomes will include the number and type of additional uterotonic drugs required, weighed estimated blood loss at delivery (in millilitres), transfusion of blood products (number of units and type of blood product), use of other surgical/mechanical measures to treat PPH (need for manual removal of the placenta/examination under anaesthetic/intrauterine balloon/uterine compression suture/interventional radiology/hysterectomy) and maternal blood pressure at 1 and 2 h postnatal.

##### Maternal acceptability outcomes

Patient-reported outcomes will include symptoms of nausea, vomiting, headache, dizziness or abdominal pain.

##### Other data collected and cost outcomes

Other data collected to allow for adjustment of results and analysis are maternal age, body mass index (BMI), ethnicity, history of past PPH, gravity, parity, onset of labour (spontaneous or induced), length of first, second and third stages of labour (in minutes), intrapartum pyrexia, weight of baby, mode of birth, gestation at birth, length of inpatient stay, use of oxytocin to induce or augment labour, use of terbutaline sulphate during labour, use of tranexamic acid, length of time spent in recovery (minutes), length of time between delivery and discharge from the labour ward (minutes), total length of the postnatal hospital stay (minutes) and maternal health-related quality of life (measured with the EQ-5D-5 L antenatally, day 1 and day 14 postpartum).

### Patient and public involvement

Patients and the public were not involved with the design, planning or delivery of the IMox study. Results of the study will not be directly communicated to study participants due to the considerable burden of doing so. However, summaries of results will be distributed using existing networks of patients (such as Maternity Voices, a maternity advocacy group within the South West of England).

### Recruitment

Eligible women will be approached by members of the research team in the study sites from 20 weeks gestation. Each woman will be provided with a Participant Information Leaflet (PIL) and invited to provide her informed consent. No trial procedures will take place before informed consent is received.

### Blinding, randomisation and allocation

Participants, members of the research team and clinical staff caring for the woman will be blinded to the allocated uterotonic. Study drugs will be manufactured in identical 1-ml vials by an independent pharmaceutical unit and block-randomised according to a computer-generated randomisation code. When vaginal birth is imminent, the next consecutively numbered study drug will be taken from the labour ward refrigerator and administered. Unblinding will be possible in emergency clinical situations where knowledge of the administered drug would change the patient’s management via a 24-h telephone service.

### Data collection methods

**Fig. 2:**
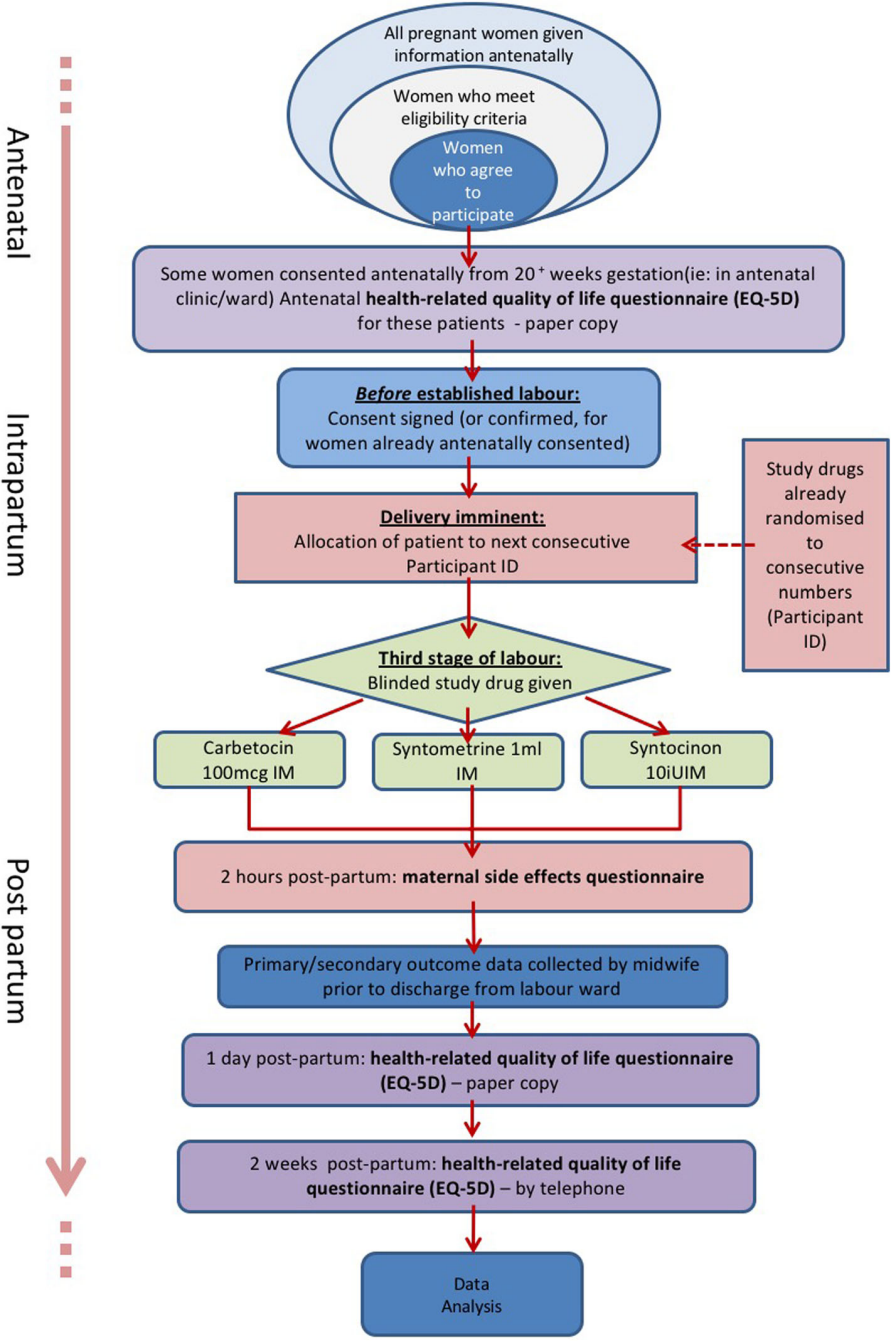
Participant timeline for the IMox study

**Fig. 3:**
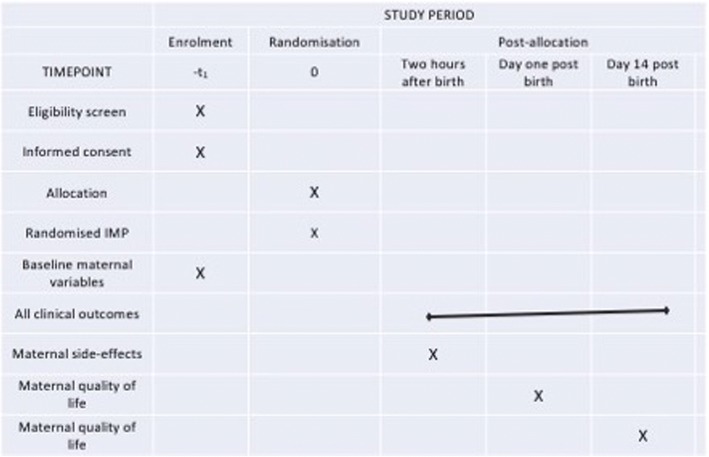
SPIRIT diagram for the IMox study

Primary and secondary outcome data will be collected before the participant is discharged from the labour ward. Maternal quality of life data will be collected in person antenatally prior to the onset of labour and on day 1 postnatally during an interview with a member of the study team. Maternal quality of life at 2 weeks postpartum will be collected via a telephone interview with the participant. If participants are not contactable by telephone, they will be sent a data collection form with a stamped, addressed envelope. A participant timeline is shown in Fig. 2. All data will be entered into an online, password protected Microsoft Access database (Microsoft Corporation, Redmond, Washington, USA) by members of the research team. The structure of the IMox study is shown in (Fig. 3).

### Sample size

The sample size selected for use in this study is 5712 participants returning data for the primary outcome. This sample size is large enough to cover all three comparisons. A drop-out rate of less than 0.5% between randomisation (birth imminent) and administration of drug is anticipated; therefore, the sample size has not been inflated.

We are investigating whether carbetocin is at least as effective as oxytocin/ergometrine and more effective than oxytocin, based on a primary outcome measure of the need for additional uterotonic drugs. Limited previous research indicates that carbetocin may be more effective than oxytocin/ergometrine at preventing PPH and is associated with significantly fewer side effects. Therefore, if carbetocin is robustly shown to be non-inferior to oxytocin/ergometrine for the primary outcome and is associated with fewer maternal side effects, it would be more appropriate for use as a prophylactic uterotonic drug.

Comparisons made will therefore be:

- Superiority comparison between oxytocin/ergometrine and oxytocin
- Superiority comparison between carbetocin and oxytocin
- Non-inferiority comparison between carbetocin and oxytocin/ergometrine

The study will be powered in accordance with European Medicines Agency guidelines and with caution exercised on the available historical data.

Broadly pooled weighted mean prevalence (weighted for study size) data on the need for additional uterotonic drugs, from the published literature comparing intramuscular uterotonics for vaginal deliveries, are shown in Table 1. Therefore, the differences between drugs equate to approximately 4 points.

**Table 1:** Prevalence of primary outcome by uterotonic drug in the published literature

	Pooled prevalence: need for subsequent additional uterotonic drugs	Percentage not requiring additional uterotonic drugs
Oxytocin	19.1%	80.9
Oxytocin/ergometrine	15.2%	84.8
Carbetocin	11.5%	88.5%

Adapted from [12, 23]

#### Superiority comparisons

To identify a 4-point difference (equivalent to a 20% reduction (15/19 = 0.8), which is clinically significant) between the current nationally recommended drug (oxytocin) and the current most commonly used drug (oxytocin/ergometrine), a sample size of 1904 per arm would provide at least 88% power for this comparison with an α of 0.05. The comparison would have a power of 80% for a Bonferroni-corrected level of nominal significance of α = 0.0167.

To identify an 8-point difference (equivalent to a 40% reduction (11/19 = 0.6), which is clinically significant) between the current nationally recommended drug (oxytocin) and the new alternative (carbetocin), the proposed sample size of 1904 per arm would have at least 99% power for this comparison either using an α of 0.05 (two-sided) or with a Bonferroni-corrected α of 0.0167.

#### Non-inferiority comparison

Non-inferiority is assessed using a two-sided CI for the difference in proportions, and significance declared should the CI lie entirely on the correct side of the non-inferiority margin. For a two-sided 95% CI, a sample size of 1904 per arm has 95% power for a non-inferiority margin of 1% using the estimated prevalence data. A Bonferroni-adjusted approach, using a two-sided 98% CI would have a power of 88% under the same scenario.

Based on the above, and in acknowledgement that the historic prevalence data may be not be precise, the trial will proceed on a target sample size of *n* = 1904 per arm. In accordance with the European Medicines Agency Guideline on Multiplicity Issues in Clinical Trials [15], an α = 0.05 will be used to determine statistical significance for all three contrasts, and additionally a threshold of α = 0.0167 (i.e. Bonferroni-corrected levels of significance) will be used for definitive statistical evidence.

### Data analysis: drug efficacy and side effects experienced by participants

The statistical analysis of the present study is performed in accordance with the principles stated in the Consensus Guideline E9 (Statistical Principles for Clinical Trials) of the International Conference on Harmonisation (ICH) [16], the guidelines in the CONSORT statement for randomised trials [17], the European Medicines Agency Guideline on Multiplicity Issues in Clinical Trials [15] and the European Medicines Agency Guideline on the Choice of the Non-Inferiority Margin [18].

A full statistical analysis plan detailing all preplanned analyses and the methods of analysis will be finalised prior to consenting the last participant. Analyses will be performed on a modified intention-to-treat basis and additionally repeated on a per protocol basis. Analysis of the primary outcome variable and additional secondary analyses, subset analyses and subgroup analyses will be described in the statistical analysis plan.

In brief, primary, secondary and safety outcome data will be reported by randomised trial arm. Continuous variables will be summarised using the mean and standard deviation (or median and interquartile range if the distribution is skewed), and categorical data will be summarised as a number and percentage.

The primary outcome variable is the need for additional uterotonic drugs. An omnibus test for differences in the proportions needing (not needing) additional uterotonic drugs will be examined using the chi-square test of association.

The chi-square test of association for a two by two cross-tabulation (equivalent to a test for a difference in two binomial proportions) will be used to examine superiority between oxytocin/ergometrine and oxytocin and between carbetocin and oxytocin. These differences will be further summarised using 95% Wald CIs for the difference between two independent proportions along with 95% CIs for the odds ratio and relative risk. Statistical significance will be declared if effects are in the anticipated direction and if the two-sided *p* value is less than 0.05. Definitive statistical significance will be declared if the *p* value is below 0.0167 (Bonferroni-adjusted level of significance). The non-inferiority comparison between carbetocin and oxytocin/ergometrine will proceed using a two-sided 95% CI for the percentage in need of additional uterotonic drugs. Non-inferiority will be declared if this interval excludes the non-inferiority margin.

Multivariable logistic regression for the primary outcome variable will also be used to adjust for known PPH risk factors: previous PPH, Asian ethnicity, obesity (BMI ≥ 35), induction of labour, operative vaginal birth, prolonged labour (≥ 12 h), big baby (≥; 4 kg), pyrexia in labour (≥ 38 °C once or ≥ 37.5 °C twice 2 h apart) and parity [5]. We will also control for clustering by centre by adjusting for maternity units as fixed effects. A PPH risk factor will only be included in the model providing missing data is less than 20%. Multiple imputation will be used to examine sensitivity of conclusions to missing data on PPH risk factors.

Analyses for all secondary outcomes and subsequent analyses will be superiority analyses. Analyses for secondary outcomes will comprise an omnibus test (parametric analysis of variance (ANOVA), Kruskal-Wallis test, chi-square test of association as appropriate) and appropriate pairwise comparisons between trial arms. Regression models will also be used to adjust for PPH risk factors. These models will be multivariable logistic regression models for binary outcomes and multivariable linear regression models (with appropriate transformation of the outcome if required) for scale outcomes.

Effect sizes and their associated 95% CIs will be calculated for each pairwise comparison.

### Cost-effectiveness analysis

Economic evaluation will be conducted from a National Health Service (NHS) secondary care perspective. Resource use will be valued using published cost data (for example from NHS Reference Costs, the Personal Social Services Research Unit (PSSRU) and the British National Formulary), as well as information obtained from each hospital’s finance department (i.e. ward costs). EQ-5D-5 L values will be converted into UK value set utilities using the ‘crosswalk’ methodology as per the National Institute for Health and Care Excellence (NICE) statement of August 2017 [19]. Quality-adjusted life years (QALYs) will be estimated using the area under the curve method [20]. Costs, QALYs and outcomes will be estimated using regression, adjusting for trial arm and stratification variables as per statistical analysis, including baseline quality of life score for QALYs.

In a cost consequences framework, we will report, per trial arm, all mean values for adjusted costs and relevant outcomes with standard deviations, including QALYs, the primary clinical outcome of need for additional uterotonic drugs and secondary outcomes of PPH, hypertension, vomiting and need for further intervention in theatre. Utility will also be analysed by level of PPH to help understand whether experiencing PPH leads to a lower utility postpartum, and for how long this effect lasts.

A separate cost-effectiveness evaluation will be performed, using effectiveness estimates directly observed in the trial data (including measures of uncertainty) and data from the utility analysis. These data will be combined to calculate the bootstrapped adjusted incremental net monetary benefit statistic and cost per QALY of the three competing treatments, for non-dominant arms. In general, a cost per QALY under £20,000 is considered a good use of NHS resources, but we will also use thresholds of £10,000 and £30,000 per QALY. We will derive cost-effectiveness acceptability curves to illustrate the uncertainty around the probability of the interventions being cost-effective for a range of thresholds.

### Adverse events

Adverse events will be assessed and reported in line with the policy of the Sponsor. Outcomes which do not need reporting include those relating to the participant’s baby (as the umbilical cord is clamped when the study drug is given), the participant ≥ 12 h after birth (as the effects of the study drug are not expected to last beyond this point) and those which occur between the time of consent and birth (before randomisation and study drug administration occurs).

### Monitoring

Monitoring will take place both remotely and on site. Site visits will take place 6–8 weeks before each site starts recruiting, 3 and 9 months into recruitment and after recruitment has finished. Additional on-site visits will be arranged to address any particular concerns which arise, or if sites require additional support. Auditing will take place when requested by the Sponsor.

### Trial oversight

The trial will be overseen by a Trial Steering Committee (TSC) and a Data Monitoring Committee (DMC). The TSC will consist of an independent Chair, the Chief Investigator and Principal Investigators from all study sites. The TSC will meet quarterly to provide oversight for the trial and ensure it is conducted in line with the principles of Good Clinical Practice (GCP).

Interim safety results and primary outcome measure were reviewed by the independent DMC after 50% of recruitment had been completed. Premature termination of the study was not recommended as emerging data (or new literature) did not show beyond reasonable doubt that any of the study drugs are clearly better for all women or subgroups.

The study is sponsored by North Bristol NHS Trust. Site-specific approvals for the conduct of the study have been obtained as required. Changes to the protocol will be implemented as required, incorporated into trial registration and communicated to study sites once secured. The Sponsor contributed to the initial study design. Neither the Sponsor (North Bristol NHS Trust) nor the funder (Ferring Pharmaceuticals Ltd.) will have any role in the trial results analysis, interpretation, writing of the report or decision to submit for publication.

The Standard Protocol Items: Recommendations for Interventional Trials (SPIRIT) checklist was used when writing this manuscript [21] and is given in Additional file 1.

### Dissemination

Study results will be published within 2 years of completion of data collection in a peer-reviewed open access journal, and the results will be presented at local, national and international meetings. Health economics results will be published separately within 2 years of completion of data collection. Summaries will also be distributed using existing networks of patients (such as Maternity Voices, a maternity advocacy group within the South West of England). A summary of results will also be sent to all units that participated in the study, unless they express the wish not to receive such information. Results will be communicated to a lay audience by social media activities of the University of Bristol, North Bristol NHS Foundation Trust and the research team. Participants will be able to register an interest in study results by emailing the study email address.

## Discussion

This is the first randomised controlled trial investigating whether carbetocin is an effective and affordable alternative to oxytocin and oxytocin/ergometrine for prevention of PPH after vaginal birth, and whether it is associated with fewer side effects for new mothers. Although this study is based in the UK, its findings will be relevant to all healthcare settings, given the global distribution of PPH. A potential limitation of this study is the choice of primary outcome of requirement for additional uterotonics. While we believe this is the most robust way to compare the real-world clinical efficacy of the studied drugs (due to the wide variations reported between observers of estimated blood loss), we acknowledge that this means our study is unlikely to be powered to determine significant differences in estimated blood loss between the studied drugs, the historically more commonly reported primary outcome. This does not however prevent the data gathered on estimated blood loss from being utilised in future meta-analyses of studies.

A core outcome set for trials examining primary prevention of PPH was published by a multinational Delphi consensus group after the commencement of the IMox study [22]. We acknowledge that this study does not include all of these outcomes. The IMox study included blood loss, maternal death, use of additional uterotonics, blood transfusion, adverse effects and transfer for higher level of care. It partially included acceptability and satisfaction with the intervention as well as women’s sense of well-being. It did not include breastfeeding.

This study does not use stratified randomisation to balance PPH risk factors across treatment arms. Doing so would have made randomisation, at the time when birth becomes imminent, logistically more difficult. The large sample size will go some way to aid balance across the arms, as will adjustment for these variables at the time of statistical analysis.

As one drug (oxytocin/ergometrine) is significantly more associated with visible side effects (nausea and vomiting) than the other studied drugs, there is potential here for practitioners to (correctly or incorrectly) impute what a participant may have received from the effect on that participant, and alter their practice accordingly. It is not possible to quantify this potential effect, and this has not been reported in previous studies which have studied oxytocin/ergometrine.

Irrespective of our findings, our study will answer the questions surrounding the use of carbetocin for vaginal birth and will contribute to the body of evidence comparing oxytocin and oxytocin/ergometrine. Moreover, this study will also provide health-related quality of life data, which do not currently exist for this part of maternity care. This study has the potential to inform and potentially improve current clinical practice as well as to optimise the birth experience of all women giving birth.

### Trial status

Authors should report the protocol version number and date, the date recruitment began and the approximate date when recruitment will be completed.

Recruitment began in February 2015 and was completed in August 2018. Protocol version 14 is currently active. This is illustrated in the IMox SPIRIT figure.

## Additional file

Additional file 1: SPIRIT checklist for IMox study. (DOCX 24 kb)

## Abbreviations

DMC: Data Monitoring Committee
PPH: Postpartum haemorrhage
QALY: Quality-adjusted life year
TSC: Trial Steering Committee

## References

[1] Say L, Chou D, Gemmill A, Tunçalp Ö, Moller A-B, Daniels J, et al. Global causes of maternal death: a WHO systematic analysis. The Lancet Global Health. 2014;2(6):e323–33. 10.1016/S2214-109X(14)70227-X 25103301

[2] Thompson JF, Roberts CL, Ellwood DA (2011). Emotional and physical health outcomes after significant primary post-partum haemorrhage (PPH): a multicentre cohort study. Aust N Z J Obstet Gynaecol.

[3] Oyelese Y, Ananth CV (2010). Postpartum hemorrhage: epidemiology, risk factors, and causes. Clin Obstet Gynecol.

[4] Begley CM, Gyte GML, Devane D, McGuire W, Weeks A (2015). Active versus expectant management for women in the third stage of labour. Cochrane Database Syst Rev.

[5] World Health Organization (2012). WHO recommendations for the prevention and treatment of postpartum haemorrhage.

[6] RCOG (2016). Prevention and Management of Postpartum Haemorrhage. BJOG.

[7] National Collaborating Centre for Women's and Children's Health (UK) (2014). Intrapartum care: care of healthy women and their babies during hildbirth.

[8] Engstrøm T, Barth T, Melin P, Vilhardt H (1998). Oxytocin receptor binding and uterotonic activity of carbetocin and its metabolites following enzymatic degradation. Eur J Pharmacol.

[9] Gallos ID, Williams HM, Price MJ, Merriel A, Gee H, Lissauer D (2018). Uterotonic agents for preventing postpartum haemorrhage: a network meta-analysis. Cochrane Database Syst Rev.

[10] Widmer M, Piaggio G, Nguyen TMH, Osoti A, Owa OO, Misra S (2018). Heat-stable carbetocin versus oxytocin to prevent hemorrhage after vaginal birth. N Engl J Med.

[11] International Confederation of Midwives, International Federation of Gynaecologists and Obstetricians (2004). Joint statement: management of the third stage of labour to prevent post-partum haemorrhage. J Midwifery Womens Health.

[12] Su L-L, Chong Y-S, Samuel M (1996). Carbetocin for preventing postpartum haemorrhage. Su L-L, editor. Vol. 284.

[13] Duthie SJ, Ven D, Yung GL, Guang DZ, Chan SY, Ma HK (1991). Discrepancy between laboratory determination and visual estimation of blood loss during normal delivery. EJOG.

[14] Bose P, Regan F, Paterson-Brown S (2006). Improving the accuracy of estimated blood loss at obstetric haemorrhage using clinical reconstructions. BJOG.

[15] European Medicines Agency (2017). Guideline on multiplicity issues in clinical trials - for publication.

[16] Lewis JA (1999). Consensus Guideline E9 (Statistical Principles for Clinical Trials). Stat Med.

[17] Schulz KF, Altman DG, Moher D, for the CONSORT Group. CONSORT 2010 Statement: updated guidelines for reporting parallel group randomised trials. BMJ. 2010;340(mar23 1):c332–2. 10.1136/bmj.c332 PMC2844940 20332509

[18] Committee for Medicinal Products for Human Use (2006). Committee for Medicinal Products for Human Use (CHMP) guideline on the choice of the non-inferiority margin. Stat Med.

[19] EuroQol (2017). National Institute for Health and Care Excellence position statement on use of the EQ-5D-5L value set.

[20] Manca A, Hawkins N, Sculpher MJ (2005). Estimating mean QALYs in trial-based cost-effectiveness analysis: the importance of controlling for baseline utility. Health Econ.

[21] Chan A-W, Tetzlaff JM, Altman DG, Laupacis A, Gøtzsche PC, Krleza-Jeric K (2013). SPIRIT 2013 statement: defining standard protocol items for clinical trials. Ann Intern Med.

[22] Meher S, Cuthbert A, Kirkham JJ, Williamson P, Abalos E, Aflaifel N (2019). Core outcome sets for prevention and treatment of postpartum haemorrhage: an international Delphi consensus study. BJOG.

[23] McDonald SJ (2004). Prophylactic ergometrine-oxytocin versus oxytocin for the third stage of labour. Cochrane Pregnancy and Childbirth Group, editor. Cochrane Database Syst Rev.

